# Does cognitive behavioral therapy for anxiety disorders assist the discontinuation of benzodiazepines among patients with anxiety disorders? A systematic review and meta‐analysis

**DOI:** 10.1111/pcn.13195

**Published:** 2021-02-25

**Authors:** Masahiro Takeshima, Tempei Otsubo, Daisuke Funada, Maki Murakami, Takashi Usami, Yoshihiro Maeda, Taisuke Yamamoto, Toshihiko Matsumoto, Takuya Shimane, Yumi Aoki, Takeshi Otowa, Masayuki Tani, Gaku Yamanaka, Yojiro Sakai, Tomohiko Murao, Ken Inada, Hiroki Yamada, Toshiaki Kikuchi, Tsukasa Sasaki, Norio Watanabe, Kazuo Mishima, Yoshikazu Takaesu

**Affiliations:** ^1^ Department of Neuropsychiatry Akita University Graduate School of Medicine Akita Japan; ^2^ Department of Psychiatry Tokyo Women's Medical University Medical Center East Tokyo Japan; ^3^ Department of Psychiatry, National Center Hospital National Center of Neurology and Psychiatry Tokyo Japan; ^4^ Department of Drug Dependence Research, National Institute of Mental Health National Center of Neurology and Psychiatry Tokyo Japan; ^5^ Psychiatric & Mental Health Nursing, Graduate School of Nursing St. Luke's International University Tokyo Japan; ^6^ Department of Psychiatry NTT Medical Center Tokyo Tokyo Japan; ^7^ Department of Psychiatry Oouchi Hospital Tokyo Japan; ^8^ Department of Psychiatry Yokohama Clinic Yokohama Japan; ^9^ Department of Psychiatry Akasaka Clinic Tokyo Japan; ^10^ Department of Psychiatry Tokyo Women's Medical University Tokyo Japan; ^11^ Department of Psychiatry Showa University Northern Yokohama Hospital Yokohama Japan; ^12^ Department of Neuropsychiatry Keio University School of Medicine Tokyo Japan; ^13^ Laboratory of Health Education, Graduate School of Education The University of Tokyo Tokyo Japan; ^14^ Department of Health Promotion and Human Behavior and of Clinical Epidemiology Kyoto University Graduate School of Medicine Kyoto Japan; ^15^ Department of Neuropsychiatry Kyorin University School of Medicine Tokyo Japan; ^16^ Department of Neuropsychiatry, Faculty of Medicine University of the Ryukyus Okinawa Japan

**Keywords:** anxiety disorder, anxiolytics, benzodiazepines, cognitive behavioral therapy, meta‐analysis

## Abstract

Long‐term use of benzodiazepines (BZD) is not recommended for the treatment of anxiety disorders. Cognitive behavioral therapy (CBT) is an effective treatment option for discontinuation of BZD in patients with anxiety disorders. This systematic review and meta‐analysis sought to clarify whether CBT is effective for discontinuing BZD anxiolytics in patients with anxiety disorders. This study was preregistered with PROSPERO (registration number: CRD42019125263). A literature search of major electronic databases was conducted in December 2018. Three randomized controlled trials were included in this review, and meta‐analyses were performed. The proportion of discontinuing BZD anxiolytics was significantly higher in the CBT plus gradual tapering group than in the gradual tapering alone group, both in the short term (3 months after allocation; number needed to treat: 3.2, 95% confidence interval [CI]: 2.1 to 7.1; risk ratio: 1.96, 95%CI: 1.29 to 2.98, *P* = 0.002, three studies) and long term (6 to 12 months after allocation; number needed to treat: 2.8, 95%CI: 1.9 to 5.3; risk ratio: 2.16, 95%CI: 1.41 to 3.32, *P* = 0.0004, three studies). CBT may be effective for discontinuing BZD anxiolytics, both in the short term and in the long term after the allocation. Further studies with larger sample sizes are necessary to draw definitive conclusions regarding the efficacy and safety of CBT for discontinuing BZD anxiolytics in patients with anxiety disorders.

Anxiety disorders are one of the most common mental disorders.^1^, ^2^ Previous studies have shown that anxiety disorders are associated with the worst mental and physical functioning^3^; suicidal ideation and suicide attempts^4^; significant impairment in personal, social, and academic functioning^5^; quality‐of‐life impairment^6^; and higher health‐care costs.^7^ In treating acute‐phase anxiety disorders, benzodiazepine (BZD) anxiolytics are one of the treatment options. However, long‐term use of BZD anxiolytics is not recommended for the treatment of anxiety disorders, as the long‐term effectiveness of BZD anxiolytics remains unclear. In addition, the long‐term use of BZD has been reported to be associated with adverse events, such as dependence,^8^ cognitive impairment,^9^ falls and fractures,^10^, ^11^ and impaired driving ability.^12^ Therefore, most guidelines recommend BZD anxiolytics only for short‐term use.^13^, ^14^, ^15^, ^16^, ^17^ Furthermore, neither the United Kingdom's National Institute for Health and Care Excellence nor the equivalent German guidelines recommend the use of BZD anxiolytics in the treatment of anxiety disorders, even for short‐term use, except for exceptional or critical situations.^18^, ^19^

In clinical practice, however, BZD anxiolytics are often used for the long‐term treatment of anxiety disorders.^20^, ^21^ An observational study in a Canadian primary‐care setting found that 22.6% of patients with an anxiety disorder used BZD and that 79.6% were long‐term BZD users, over the course of 180 days.^20^ Another observational study conducted at clinical treatment facilities in the USA that followed patients with anxiety disorders for 12 years showed that 55.7% of patients with generalized anxiety disorder, 47.4% of patients with social anxiety disorder, and 57.9% of patients with panic disorder continued taking BZD throughout the study period.^21^, ^22^ Thus, the development of a treatment strategy against the long‐term use of BZD in patients with anxiety disorders may be warranted in clinical settings.

Cognitive behavioral therapy (CBT) is an effective treatment option for anxiety disorders.^23^, ^24^ Several guidelines recommend it as a first‐line treatment, because of its efficacy in improving anxiety symptoms and minimal adverse effects compared to pharmacological treatments.^13^, ^15^, ^19^ In addition, a Cochrane review demonstrated the effectiveness of CBT on BZD discontinuation for long‐term BZD users with coexisting anxiety disorders, chronic insomnia, or BZD dependence, at least in the short term.^25^ However, the Cochrane review did not focus on the effectiveness of CBT in discontinuing BZD in patients with anxiety disorders^25^; hence, it remains unclear whether CBT is effective in discontinuing BZD in patients with anxiety disorders.

Therefore, we sought to conduct a systematic review and meta‐analysis to clarify whether CBT is effective as a tool toward discontinuing BZD anxiolytics in patients with anxiety disorders. We also sought to investigate the advantages of improving the severity of anxiety symptoms, as compared to the simple gradual tapering of BZD anxiolytics in the short and long term.

## Methods

This study was conducted in accordance with the Preferred Reporting Items for Systematic Reviews and Meta‐Analyses (PRISMA) recommendations for reporting systematic reviews and meta‐analyses^26^ and preregistered with PROSPERO (https://www.crd.york.ac.uk/PROSPERO/#searchadvanced, CRD42019125263).

### Search strategy

We searched the PubMed electronic databases (search date: 12 October 2018), Cochrane Central Register of Controlled Trials (CENTRAL; search date: 7 November 2018), Embase (search date: 5 December 2018), and ClinicalTrials.gov (search date: 12 October 2018) for reports of randomized controlled trials (RCT), using appropriate subject headings and search syntaxes that were relevant to each resource (e.g., ‘anxiety disorder,’ ‘cognitive behavioral therapy,’ and ‘taper’; Table S1).

### Inclusion criteria

Studies in any language that met the following criteria were included in the final review:

1. Participants diagnosed with an anxiety disorder (panic disorder, social anxiety disorder, generalized anxiety disorder, or specific phobia) according to diagnostic criteria (DSM‐III, DSM‐III‐R, DSM‐IV, DSM‐IV‐TR, DSM‐5, and ICD‐10).
2. Participants taking BZD anxiolytics.
3. Participants taking BZD anxiolytics at least 4 days a week, for at least 3 months.
4. Participants aged 18 years or over.
5. Participants with no signs of dementia, substance dependence, schizophrenia, or intellectual disability.
6. Interventions included gradual tapering of BZD anxiolytics, plus one‐to‐one structured CBT performed by trained staff aimed at treating anxiety symptoms. Relaxation or mindfulness alone was not considered to constitute CBT. CBT includes not only face‐to‐face interventions, but also telephone, computer, and virtual reality interventions.
7. Participants were randomly allocated in a minimal intervention group, undergoing a gradual tapering of BZD anxiolytics alone or through a relaxation program, as a control condition.
8. Pre‐ and post‐treatment data were provided for both intervention and control groups for information regarding the number of BZD anxiolytics.
9. Studies were carried out as RCT.
10. The research period exceeded 3 months.

### Article selection process

Author Ta.U. removed duplicates. Subsequently, six groups to which two authors belonged were created (Ta. U. and Ma. M., Ta. Y. and Ma. M., Da. F. and Ta. U., Ta. U. and Ta. Y., and Ma. M. and Da. F.). In each group, the two authors independently screened the titles and abstracts of the identified references to exclude irrelevant studies. Five groups of two authors were then created (Da. F. and Yo. M., Da. F. and Ma. M., Ta. Y. and Yo. M., Ta. U. and Ta. Y., and Ta. U. and Yo. M.). The full texts of these references were evaluated, and ineligible reports were excluded according to the above criteria. The reasons for exclusion were registered by the authors in each group. Any disagreement was resolved by systematic and thorough discussion with another author (Yo. T.).

### Outcome measures

The primary outcome measures were the proportions of BZD anxiolytics discontinuation in the short term (up to 3 months after the allocation) and long term (6 to 12 months after the allocation). In addition, we assessed improvements in the severity of anxiety symptoms in the short and long term after the allocation, as well as the dropout proportion for any reason in the long term, as secondary outcomes. Anxiety symptoms were evaluated using the following scales in order of prioritization: Beck Anxiety Inventory, Generalized Anxiety Disorder 7‐item Scale, and the Penn State Worry Questionnaire. For example, if a study used both the Beck Anxiety Inventory and the Generalized Anxiety Disorder 7‐item Scale, the results of the Beck Anxiety Inventory were selected for assessment of the severity of anxiety symptoms. When a three‐arm study included two different control groups, we selected only one control group with gradual BZD tapering.

### Study quality and risk‐of‐bias assessment

Ma. T. and Yo. T. independently extracted the data, and Te. O. performed checks to ensure their accuracy. The following variables were recorded: participant characteristics, diagnostic criteria of the anxiety disorder, study design, details of the treatment component, treatment duration, control intervention, and outcome measures. The quality of each included study was evaluated by the Ma. T. and Yo. T. groups using the Cochrane risk‐of‐bias assessment.^27^ The assessment evaluates RCT in seven domains, including random‐sequence generation, allocation concealment, blinding of participants, personnel and outcome assessors, incomplete outcome data, selective outcome reporting, and other sources of bias. Selective outcome reporting was defined by whether the trial was analyzed and reported in accordance with a prespecified plan that was finalized before unblinded outcome data were available for analysis. Other bias was defined as potential unit‐of‐analysis bias due to cluster RCT design. The rating of each domain can be ‘yes’ (low risk of bias), ‘no’ (high risk of bias), or ‘unclear’ (uncertain risk). Disagreements were resolved by systematic and thorough discussions with Te. O.

### Statistical analyses

We used the Cochrane Collaboration Review Manager software (RevMan 5.3; https://training.cochrane.org/online-learning/core-software-cochrane-reviews/revman) for statistical analysis. Continuous outcome data were summarized using effect size, with ‘standardized mean differences,’ with 95% confidence intervals (CI); for dichotomous outcomes, risk ratios with 95%CI were used. We used random effects models for data analyses. For cases where the risk ratios showed statistically significant differences between intervention and control groups, the number needed to treat (NNT) or number needed to harm (NNH) was calculated from the risk difference, using the formula NNT or NNH = 1/risk difference. Publication bias was evaluated using a funnel plot of treatment effect against a standard error and Egger's test when at least 10 studies were available.^28^ Assessments of treatment adherence, acceptability, perceived utility, and credibility were reviewed.

## Results

### Description of studies included for review

**Fig. 1:**
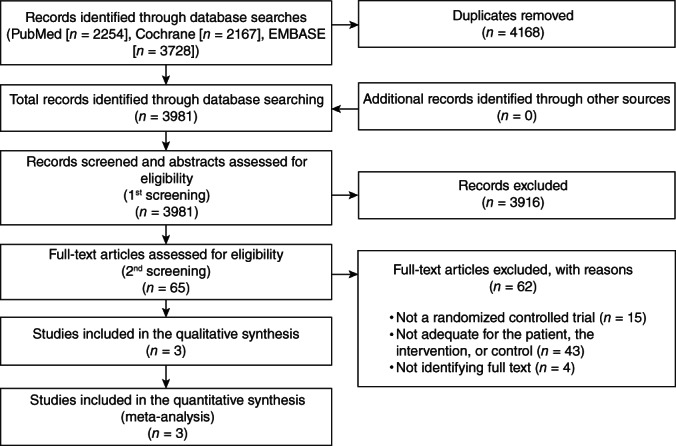
Flowchart of the study selection process for the studies included in the review.

The initial literature search yielded 3981 results after exclusion of duplicates (PubMed = 2254, CENTRAL = 2167, Embase = 3728) up to December 2018. No ongoing clinical trial was identified on ClinicalTrails.gov up to October 2018. After reading the titles and abstracts of the identified reports, a total of 65 were retrieved in full‐text, whereas 62 were excluded for various reasons (Table S2). The remaining three RCT were included in this review (Fig. 1).

### Study characteristics

Three articles consisting of three studies published between 1994 and 2010 were included in this review.^29^, ^30^, ^31^ The sample sizes of the three studies ranged from 21 to 61, with a total of 113 participants (Table 1). Of all participants, 65.5% were female, and the participants' mean age was 45.8 years. All participants had anxiety disorders. One study was focused on panic disorders with agoraphobia,^31^ another study was focused on generalized anxiety disorder,^30^ and a further study was focused on panic disorders with or without agoraphobia.^29^ The criteria used for the diagnosis of anxiety disorders varied across studies. Two studies used the Anxiety Disorders Interview Schedule (ADIS) for the DSM‐IV,^32^ and one study used the ADIS‐revised version.^33^ One study excluded major mood disorders,^31^ and two studies excluded bipolar disorder.^29^, ^30^ All of the subjects included in this review took BZD anxiolytics and none had previously undergone CBT for any of the anxiety disorders targeted in the studies. Two studies used antidepressants.^29^, ^30^ One study did not allow for any concomitant psychotropic medication other than BZD anxiolytics.^31^

**Table 1:** Characteristics of the study participants

Study (year)	Enrolled patients (mean age)^†^	Female	Study design of RCT	Diagnosis	Diagnostic criteria for anxiety disorder	Concomitant psychiatric disorders, *n* (%)	Duration of taking BZD (mean ± SD)	Concomitant psychotropic drugs	Country
Spiegel *et al*. (1994)^31^	21 adults (38.0 years)	81.0% (17/21)	Two‐arm CBT = 11 TAU = 10	PD with agoraphobia	ADIS‐Revised	Other anxiety disorder 57% GAD 33% Simple phobia 29% Social phobia 9% Personality disorder 33%	Range 14 to 592 weeks (93.4 ± 149.4 weeks)	None	USA
Gosselin *et al*. (2006)^30^	61 adults (50.3 years)	59.0% (36/61)	Two‐arm CBT = 31 TAU = 30	GAD	ADIS for DSM‐IV	Social phobia 44.3% (27/61) Specific phobias 31.1% (19/61) Panic disorder 18.0% (11/61) Major depression 16.4% (10/61) Insomnia 6.6% (4/61) Dysthymic disorder 4.9% (3/61) PTSD 3.3% (2/61) OCD 1.6% (1/61).	More than 12 months (7.25 ± 5.95 years)	Psychotropic drugs other than BZD 47.5% (29/61)	Canada
Otto *et al*. (2010)^29^	31 adults (42.3 years)	67.7% (21/31)	Three‐arm CBT = 16 IRT = 16 TAU = 15	PD with or without agoraphobia	ADIS for DSM‐IV	Comorbid anxiety disorder 43.8% (7/16) vs 60% (9/15) [51.6% (16/31)] Comorbid depressive disorder 56.3% (9/16) vs 53.3% (8/15) [54.8% (17/31)]	More than 6 months (4.20 ± 2.99 years)	Antidepressants 25.8% (8/31)	USA

ADIS, Anxiety Disorders Interview Schedule; BZD, benzodiazepines; CBT, cognitive behavioral therapy; GAD, generalized anxiety disorder; IRT, individual relaxation treatment; OCD, obsessive–compulsive disorder; PD, panic disorder; PTSD, post‐traumatic stress disorder; RCT, randomized controlled trial; TAU, treatment as usual.

^†^ Number of patients meeting inclusion criteria, enrolled in the study at baseline.

All three studies consisted of individual RCT conducted at a secondary‐care facility.^29^, ^30^, ^31^ Two studies consisted of two‐arm studies,^30^, ^31^ and one study was a three‐arm study.^29^ As this three‐arm study involved two separate comparisons, the total number of comparisons was greater than the number of studies included in this review. When a three‐arm study consisted of two different interventions, we selected only one intervention, which was consistent with gradual tapering alone. All studies had received public research funding.^29^, ^30^, ^31^

The treatment components for respective CBT were almost the same across all of the studies (Table 2). Across the three studies, all provided a multicomponent of individual CBT programs, consisting of psychoeducation, cognitive restructuring, and exposure.^29^, ^30^, ^31^ One study used CBT to treat panic disorders in the form of 12 weekly sessions (the duration of each session was not available) provided by two psychology graduate students and a social worker.^31^ The second study used CBT to treat panic disorders in the form of 12 weekly sessions each lasting 65 to 70 min, provided by psychologists.^30^ The third study used CBT to treat generalized anxiety disorders in the form of eight weekly sessions each lasting 60 min, except for the initial 90‐min session. These sessions were provided by licensed and unlicensed postdoctoral clinical staff.^29^ The study had three ‘booster’ sessions at 2, 4, and 6 weeks, after the first 8 weeks.^29^ The number of CBT sessions actually undertaken was not described in any of the three studies.

**Table 2:** Description of cognitive behavioral therapy for insomnia interventions

Study (year)	Referral	Provider of intervention (CBT)	CBT training provided	Treatment (intervention) vs control	Components of CBT	Treatment fidelity measures	No. of sessions	Duration of session	Time frame of the program
Spiegel *et al*. (1994)^31^	Patients referred to the clinic	Two psychology graduate students and a social worker	Under direct supervision of an experienced clinical psychologist	CBT + tapering vs tapering	Psychoeducation for panic disorder Diaphragmatic breathing exercise Cognitive restructuring Interoceptive exposure	Treatment‐adherence scales were used for the assessment of recorded CBT sessions. Medication adherence was assessed by pill counts, patient diaries of medication use, and serum benzodiazepine levels.	12	NA	12 weeks
Gosselin *et al*. (2006)^30^	Media advertisements	Psychologists experienced in treating anxiety disorders	Receiving weekly clinical supervision	CBT + tapering vs tapering	Psychoeducation for anxiety Cognitive restructuring Problem‐solving training Cognitive and situational exposure	Interventions were in accordance with the developed treatment manuals. Audiotaped sessions were assessed by independent psychologists.	12	65–70 min	12 weeks
Otto *et al*. (2010)^29^	Individuals who contacted the clinic	Licensed and unlicensed postdoctoral clinical staff	Highly trained in a specialty clinic of a large teaching hospital with experience in the administration of CBT	CBT + tapering vs tapering	Psychoeducation for panic disorder Cognitive restructuring Interoceptive exposure Somatic coping skills	NA	8 + 3 booster sessions	60 min, except for the initial 90‐min session	8 weeks

CBT, cognitive behavioral therapy; NA, not available.

### Risk‐of‐bias assessment

**Fig. 2:**
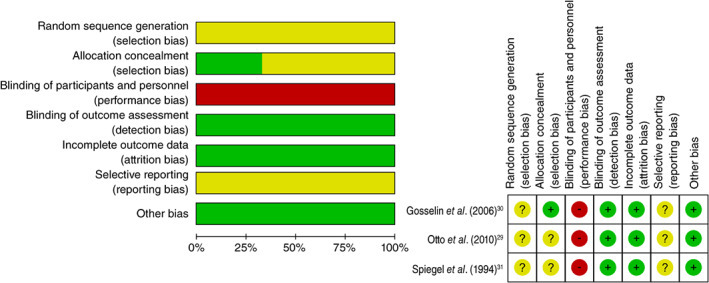
Risk‐of‐bias assessment summary. (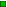) Low risk of bias. (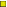) Unclear risk of bias. (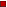) High risk of bias.

From the risk‐of‐bias summary (Fig. 2), none of the RCT reported an adequate randomization method or a sufficient allocation concealment procedure. All RCT were judged to have had a high risk of bias with regard to participant and personnel blinding due to the nature of these studies. They were judged to have had a low risk of bias with regard to blinding assessors and attrition.^29^, ^30^, ^31^ Reporting bias was unclear in all three studies because we did not obtain the research registrations of these studies.^29^, ^30^, ^31^ There was no potential unit‐of‐analysis bias as all three studies had an individual RCT design.^29^, ^30^, ^31^

### Treatment outcome assessment

The outcomes are summarized in Table 3. The proportion of discontinuation of BZD anxiolytics after the allocation was the main primary outcome measure reported across all three RCT.^29^, ^30^, ^31^ One study examined the proportion of discontinuation of BZD anxiolytics at 12 months after the allocation,^30^ whereas the other two studies examined it at 6 months after the allocation.^29^, ^31^ One study measured the severity of anxiety symptoms at both baseline and post‐allocation (the Penn State Worry Questionnaire and the Worry and Anxiety Questionnaire Somatic subscale),^30^ another study measured this at post‐allocation,^29^ and the other study did not examine it throughout the study period.^31^ Only one study examined the scores of the severity of anxiety symptoms after allocation.^30^ Regarding adverse events, one study reported that one patient in the CBT group and one patient in the control group had developed depressive symptoms requiring special interventions.^30^

**Table 3:** Summary of the outcomes

Study (year)	BZD discontinuation at 3‐month post‐allocation (intervention vs control)^†^	BZD discontinuation at 6‐ to 12‐month post‐allocation (intervention vs control)^†^	Severity of anxiety symptoms at 3‐month post‐allocation (intervention vs control)^†^	Severity of anxiety at 6‐ to 12‐month post‐allocation (intervention vs control)^†^
Spiegel *et al*. (1994)^31^	At 3 months: BZD‐free (81.8% [9/11] vs 40% [4/10], NS)	At 6 months: BZD‐free (81.8% [9/11] vs 40% [4/10], NS)	At 3 months: NA	At 6 months: NA
Gosselin *et al*. (2006)^30^	At 3 months: ↓BZD‐free (67.7% [21/31] vs 33.3% [10/30], *P* < 0.05)	At 12 months: ↓BZD‐free (64.5% [20/31] vs 30.0% [9/30], *P* < 0.05)	At 3 months: ↓PSWQ (47.6 ± 9.5 [*n* = 27] vs 53.7 ± 7.9 [*n* = 27], *P* < 0.05)	At 12 months: ↓PSWQ (44.9 ± 10.3 [*n* = 28] vs 52.8 ± 9.0 [*n* = 28], *P* < 0.05)
Otto *et al*. (2010)^29^	At 3 months: BZD‐free (43.7% [7/16] vs 26.7% [4/15], NS)	At 6 months: BZD‐free (62.5% [10/16] vs 26.7% [4/15], NS)	At 3 months: BAI (NA)	At 6 months: BAI (NA)

BAI, Beck Anxiety Inventory; BZD, benzodiazepines; NA, not available; NS, not significant; PSWQ, Penn State Worry Questionnaire.

^†^ Arrows indicate the effect on the difference between baseline and post‐intervention. Values in parentheses indicate statistical analysis results of variables before and after intervention and are indicated in the following order: (i) mean ± standard deviation (intervention vs control); (ii) *n* (%) (intervention vs control); (iii) *P*‐value.

**Fig. 3:**
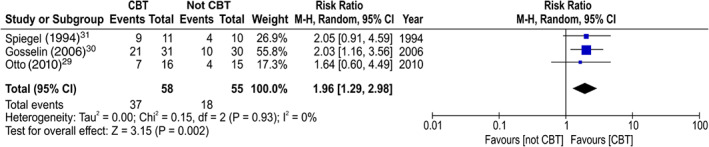
Forest plot of the treatment effect sizes for the proportion of discontinuation of benzodiazepine anxiolytics at 3 months post‐allocation. CBT, cognitive behavioral therapy; CI, confidence interval.

**Fig. 4:**
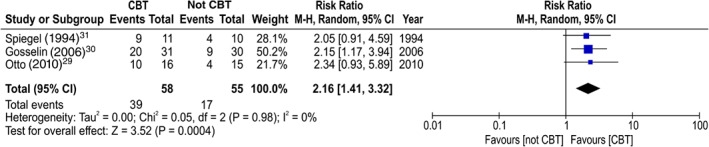
Forest plot of the treatment effect sizes for the proportion of discontinuation of benzodiazepine anxiolytics at 6 to 12 months post‐allocation. CBT, cognitive behavioral therapy; CI, confidence interval.

**Fig. 5:**
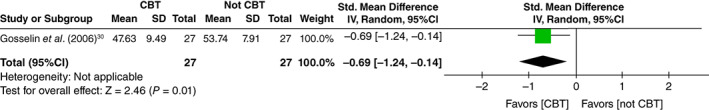
Forest plot of treatment effect sizes for anxiety symptoms at 3 months post‐allocation. CBT, cognitive behavioral therapy; CI, confidence interval.

**Fig. 6:**
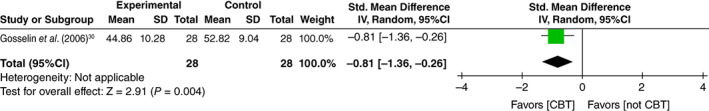
Forest plot of treatment effect sizes for anxiety symptoms at 6 to 12 months post‐allocation. CBT, cognitive behavioral therapy; CI, confidence interval.

**Fig. 7:**
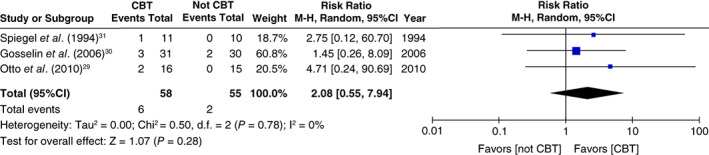
Forest plot of intervention effect sizes for dropout proportions for any reason at 6 to 12 months post‐allocation. CBT, cognitive behavioral therapy; CI, confidence interval.

The proportion of discontinuation of BZD anxiolytics at 3 months after the allocation in the CBT group was significantly higher than that in the gradual tapering (control) group (NNT: 3.2, 95%CI: 2.1 to 7.1; risk ratio: 1.96, 95%CI: 1.29 to 2.98, *P* = 0.002; 113 participants, three studies; Fig. 3).^29^, ^30^, ^31^ Regarding long‐term outcomes (6 to 12 months after the allocation), the proportion of discontinuation of BZD anxiolytics in the CBT group was also significantly higher than that in the gradual tapering (control) group (NNT: 2.8, 95%CI: 1.9 to 5.3; risk ratio: 2.16, 95%CI: 1.41 to 3.32, *P* = 0.0004; 113 participants, three studies; Fig. 4).^29^, ^30^, ^31^ The effect of anxiety symptoms could not be meta‐analyzed because only one study examined the severity scores of anxiety symptoms after allocation.^30^ Although not a result of the meta‐analysis, treatment of the anxiety symptoms in the CBT group was significantly higher than that in the gradual tapering (control) group, both in the short term (standard mean difference [SMD]: −0.69, 95%CI: −1.24 to −0.14, *P* = 0.01; 54 participants, one study) and in the long term (SMD: −0.81, 95%CI: −1.36 to −0.26, *P* = 0.004; 56 participants, one study; Figs 5 and 6).^30^ No significant differences were found in the dropout proportions between intervention and control groups (risk ratio: 2.08, 95%CI: 0.55 to 7.94, *P* = 0.28; 113 participants, three studies; Fig. 7).^29^, ^30^, ^31^

## Discussion

This is the first systematic review and meta‐analysis to evaluate the efficacy of CBT in discontinuing BZD anxiolytics for patients with anxiety disorders. The results of this review suggest that CBT is effective for discontinuing BZD anxiolytics in the long term (6 to 12 months after the allocation), as well as in the short term (up to 3 months after the allocation), alongside being effective for improving anxiety symptoms both in the short and long term. Furthermore, there was no difference in the dropout proportions between the CBT and control groups.

A Cochrane review investigated the effectiveness of psychosocial interventions, including CBT and other low‐intensive interventions, such as motivational interviewing, tailored general practitioner letters, standardized interviews, and relaxation, in discontinuing BZD.^25^ The Cochrane review reported that only CBT was robust among psychosocial interventions in discontinuing BZD, at least in the short term (up to 3 months). However, the effect of CBT in discontinuing BZD was not maintained at 6 months among long‐term BZD users.^25^ One possible reason for these discrepancies between the results of our study and the Cochrane review, in terms of long‐term effectiveness, may be related to the differences in target populations. The Cochrane review analyzed long‐term BZD users with various complicated psychiatric disorders, such as BZD dependence with or without alcohol dependence,^34^ anxiety disorders,^29^, ^30^, ^35^ and insomnia,^35^, ^36^, ^37^ at the same time. This heterogeneity of the target sample might obscure the long‐term effectiveness of CBT in discontinuing BZD.

Another systematic review and meta‐analysis focusing on chronic insomnia reported that CBT for insomnia (CBT‐I) showed a significant effect in discontinuing BZD in the short term (up to 3 months), but did not show a significant effect in the long term (6 to 12 months) in patients with chronic insomnia.^38^ With regard to the difference in the long‐term effectiveness of discontinuing BZD between our study and the CBT‐I review,^38^ this inconsistency could be due to the difference in intensity between CBT for anxiety disorder and CBT‐I. Usually, CBT‐I includes four to eight sessions, whereas CBT for anxiety disorders usually includes more than 10 sessions. This disparity may incur differences in the long‐term effectiveness of discontinuing BZD. Interestingly, in this CBT‐I review, the RCT that included two booster sessions after eight sessions of CBT‐I showed a significant effect in discontinuing BZD hypnotics in the long term.^36^ In addition to the number of sessions, the differences in the content of CBT could have caused the difference in the long‐term effectiveness of discontinuing BZD. Exposure technique is one of the most effective interventions to alleviate anxiety and related symptoms^39^ and is usually included in CBT for anxiety, but not in CBT‐I. Furthermore, a previous cohort study indicated that BZD anxiolytics had a lower risk of long‐term BZD use than BZD hypnotics,^31^ which might incur a difference in the long‐term effectiveness of discontinuing BZD between our results, focusing on BZD anxiolytics, and the previous review, focusing on BZD hypnotics. In light of these results, the homogeneity of the target sample, intensity of CBT (higher number of sessions or adjunctive booster sessions), and differences in BZD anxiolytics and hypnotics might contribute to the long‐term effectiveness of discontinuing BZD.

In this study, CBT significantly improved the severity of anxiety symptoms both in the short term and in the long term after the allocation. This result was consistent with those of previous studies, which demonstrated the long‐term effectiveness of CBT for anxiety symptoms in patients with anxiety disorders.^40^ Although it remains unclear whether the long‐term effectiveness of CBT for anxiety symptoms was associated with the long‐term effectiveness of discontinuing BZD anxiolytics, previous studies have implied a relationship between anxiety symptoms and the long‐term use of BZD. A previous 8‐year longitudinal cohort study revealed that severe anxiety symptoms were associated with inappropriate BZD use, mainly the long‐term use of BZD.^41^ Another 2‐year longitudinal study reported that the severity of anxiety predicted the long‐term use of BZD.^42^ These studies suggest that the long‐term effectiveness of CBT for anxiety symptoms might contribute to preventing the long‐term reuse of BZD anxiolytics.

### Limitations

There were several limitations to our study. First, the sample size was relatively small due to the small number of studies included in our analysis. Most of the studies identified by the literature search involved patients with anxiety disorders who were not taking BZD. Although long‐term use of BZD has been reported in several countries,^20^, ^21^ few studies have examined the effects of CBT for BZD discontinuation. Second, because we included different anxiety disorders, it would have been best to conduct a subgroup analysis for each anxiety disorder. However, we could not conduct these subgroup analyses because the studies included only three RCT (panic disorder: two RCT; generalized anxiety disorder: one RCT). Third, because the included RCT followed the effects of CBT for up to 12 months, it remains unclear whether the effects of CBT will last longer than 12 months. Fourth, performance bias may have affected the results of this study because of the inability to conceal the allocation to the patients. Fifth, all three studies included in this systematic review were included in a 2015 Cochrane review by Darker *et al*.,^25^ and we could not add new studies that were not included in the Cochrane review and analyze them. Further studies with larger sample sizes and longer evaluation periods will be necessary to draw conclusions regarding the long‐term efficacy of CBT toward discontinuing BZD anxiolytics in patients with anxiety disorders.

### Conclusions

The results of our systematic review and meta‐analysis point to the significant effects of CBT in discontinuing BZD anxiolytics for patients with anxiety disorders in both the long and short term. Our results can help physicians and patients who are willing to discontinue BZD anxiolytics in a clinical setting.

## Disclosure statement

Daisuke Funada, Maki Murakami, Takashi Usami, Yoshihiro Maeda, Taisuke Yamamoto, Toshihiko Matsumoto, Takuya Shimane, Yumi Aoki, Takeshi Otowa, Masayuki Tani, Gaku Yamanaka, Yojiro Sakai, Tomohiko Murao, Hiroki Yamada, and Norio Watanabe declare no conflict of interest. Masahiro Takeshima has received personal fees from Daiichi Sankyo Company and Meiji Seika; and grants from SHIONOGI & CO., LTD., Otsuka Pharmaceutical, and Eisai, outside the submitted work. Tempei Otsubo has received personal fees from Eli Lily, Takeda Pharmaceutical, Otsuka Pharmaceutical, Yoshitomi Yakuhin, Sumitomo Dainippon Pharma, Mochida, Meiji Seika Pharma, and Kyowa Pharmaceutical; and grants from Eisai and Otsuka Pharmaceutical, outside the submitted work. Ken Inada has received personal fees from Dainippon Sumitomo Pharma Co., Ltd., Mochida Pharmaceutical Co., Ltd., Takeda Pharmaceutical Company Limited, Novartis Pharma K.K, Meiji Seika Pharma Co., Ltd., Shionogi & Co., Ltd., Eli Lilly Japan K.K., Astellas Pharma Inc., Otsuka Pharmaceutical, Co., Ltd., Chugai Pharmaceutical Co., Ltd., Lundbeck Japan K.K., Eisai Co., Ltd., and Janssen Pharmaceutical K.K.; and grants and personal fees from MSD K.K. and Mitsubishi Tanabe Pharma Co., outside the submitted work. Toshiaki Kikuchi has received personal fees from Dainippon, Pfizer, Takeda, Eli Lilly, Mochida, Lundbeck, Otsuka, Kyowa Hakko Kirin, Meiji, Yoshitomi Yakuhin, and MSD, outside the submitted work. Tsukasa Sasaki has received personal fees from Mochida Pharmaceutical, outside the submitted work. Kazuo Mishima has received grants from the Japanese Ministry of Health, Labour, and Welfare and Eisai Co., Ltd., during the conduct of the study; personal fees from MSD Inc.; grants and personal fees from Mitsubishi Tanabe Pharma Corporation (Yoshitomiyakuhin Corporation), Takeda Pharmaceutical Co., Ltd., Nobelpharma Co., Ltd., and Otsuka Pharmaceutical Co., Ltd.; and personal fees from Astellas Pharma Inc. and Pfizer Inc., outside the submitted work. Yoshikazu Takaesu has received grants and personal fees from Otsuka Pharmaceutical, Meiji Seika, MSD, and Eisai; and personal fees from Eli Lilly, Mitsubishi Tanabe Pharma, Yoshitomi Pharmaceutical and Takeda Pharmaceutical, outside the submitted work.

## Author contributions

Ma. T. was responsible for the literature screening and wrote the Abstract, Methods, Results, and Discussion of this manuscript. Te.O. wrote the Abstract and provided important comments on the Discussion of this manuscript. Da. F., Ma. M., Ta. U., and Ta. Y. made the study plan and were responsible for the literature screening. Yo. M. was responsible for the literature screening. To. M., Ta. S., Ta. O., Ga. Y., and Ts. S. took part in the literature screening. Yu. A., Yo. S., Ma. T., To. M., Ke. I., Hi. Y., No. W., and Ka. M. provided important comments on the Discussion of this manuscript. To. K. helped with the presentation of the current evidence on CBT for anxiety disorders. Yo. T., the corresponding author, takes responsibility for collecting all information, for implementing all ideas contributed by the other authors, and for the final revision and submission of the manuscript.

## Supporting information


**Table S1**. Search strategies.

**Table S2**. List of excluded articles.
