# Supplementary material for: Does cognitive behavioral therapy for anxiety disorders assist the discontinuation of benzodiazepines among patients with anxiety disorders? A systematic review and meta‐analysis
Source: Psychiatry Clin Neurosci. 2021 Feb 25;75(4):119–27. doi: 10.1111/pcn.13195 (PMC8048602; doi:10.1111/pcn.13195)
Supplement: Supplementary file 1 — Table S1. Search strategies. [file PCN-75-119-s002.docx]

**Supporting Information**

**Supporting Table S1. Search strategies**

We searched the databases, PubMed, CENTRAL, and Embase, using the following search terms:

**PubMed**

1. MeSH descriptor: [Anxiety Disorders] explode all trees
2. anxiety disorder or anxiety or phobi* or panic or agoraphobia or chronic or "Substance Withdrawal Syndrome"
3. 1 or 2
4. MeSH descriptor: [Benzodiazepines] explode all trees
5. Benzodiazepine* or BZD or chlordiazepoxide or diazepam or alprazolam or lorazepam or prazepam or clobazam or bromazepam or flurazepam or clonazepam or oxazepam or lofrazapate or flutoprazepam or medazepam or oxazolam or flutazolam or etizolam or mexazolam or clotiazepam or tofisopam
6. 4 or 5
7. MeSH descriptor: [Behavior Therapy] explode all trees
8. MeSH descriptor: [Cognitive Therapy] explode all trees
9. ("behaviour therapy" or ("behavior" and "therapy") or "behavior therapy") or (("cognitive" and "therapy") or "cognitive therapy") or ("cognitive behaviour therapy" or ("cognitive" and "behavior" and "therapy") or "cognitive behavior therapy")
10. 7 or 8 or 9
11. abstinen* or abstain* or cessat* or detox* or discontinu* or reduce* or reducing or reduct* or stop* or taper* or withdraw* or substitut*
12. 3 and 6
13. 10 or 11
14. 12 and 13
15. Review
16. Trial

**CENTRAL**

1. ("anxiety disorders"[Mesh] OR anxiety disorder[All Fields]) OR (anxiety[All Fields] OR phobi* [All Fields] OR panic[All Fields] OR agoraphobia[All Fields] OR chronic[All Fields]) OR ("Substance Withdrawal Syndrome"[All Fields])
2. ("Benzodiazepines"[Mesh]) OR (Benzodiazepine*[All Fields] OR BZD[All Fields] OR chlordiazepoxide[All Fields] OR diazepam[All Fields] OR alprazolam[All Fields] OR lorazepam[All Fields] OR prazepam[All Fields] OR clobazam[All Fields] OR bromazepam[All Fields] OR flurazepam[All Fields] OR clonazepam[All Fields] OR oxazepam[All Fields] OR lofrazapate[All Fields] OR flutoprazepam[All Fields] OR medazepam[All Fields] OR oxazolam[All Fields] OR flutazolam[All Fields] OR etizolam[All Fields] OR mexazolam[All Fields] OR clotiazepam[All Fields] OR tofisopam[All Fields]) ((Mani$ or major) and (depres$ or disorder$))
3. ("behavior therapy"[MeSH] OR "behaviour therapy"[All Fields] OR ("behavior"[All Fields] AND "therapy"[All Fields]) OR "behavior therapy"[All Fields]) OR ("cognitive therapy"[MeSH] OR ("cognitive"[All Fields] AND "therapy"[All Fields]) OR "cognitive therapy"[All Fields]) OR ("cognitive behaviour therapy"[All Fields] OR ("cognitive"[All Fields] AND "behavior"[All Fields] AND "therapy"[All Fields]) OR "cognitive behavior therapy"[All Fields])
4. (abstinen*[All Fields] OR abstain*[All Fields] OR cessat*[All Fields] OR detox*[All Fields] OR discontinu*[All Fields] OR reduce*[ All Fields] OR reducing[All Fields] OR reduct* [All Fields] OR stop*[All Fields] OR taper*[All Fields] OR withdraw*[All Fields] OR substitut*[All Fields])
5. ((randomized controlled trial [pt] OR controlled clinical trial [pt] OR randomized [tiab] OR placebo [tiab] OR clinical trials as topic [mesh: noexp] OR randomly [tiab] OR trial [ti]) NOT (animals [mh] NOT humans [mh]))
6. 1 and 2
7. 3 or 4
8. 6 and 7
9. 5 and 8

**Embase**

1. anxiety disorder'/exp OR 'anxiety disorder' OR 'anxiety disorders'
2. anxiety':ti,ab,kw
3. phobi*':ti,ab,kw
4. panic':ti,ab,kw
5. chronic':ti,ab,kw
6. withdrawal syndrome':ti,ab,kw
7. 1 OR 2 OR 3 OR 4 OR 5 OR 6
8. benzodiazepine derivative'/exp OR '1, 4 benzodiazepin derivative' OR '1, 4 benzodiazepine derivative' OR '1, 5 benzodiazepine derivative' OR '2, 3 benzodiazepine derivative' OR '2, 4 benzodiazepine derivative' OR 'benzodiazepin derivative' OR 'benzodiazepine derivative' OR 'benzodiazepines' OR 'benzodiazepinones'
9. benzodiazepine*':ti,ab,kw
10. bzd':ti,ab,kw
11. chlordiazepoxide':ti,ab,kw
12. diazepam':ti,ab,kw
13. alprazolam':ti,ab,kw
14. lorazepam':ti,ab,kw
15. prazepam':ti,ab,kw
16. clobazam':ti,ab,kw
17. bromazepam':ti,ab,kw
18. flurazepam':ti,ab,kw
19. clonazepam':ti,ab,kw
20. oxazepam':ti,ab,kw
21. lofrazapate':ti,ab,kw
22. flutoprazepam':ti,ab,kw
23. medazepam':ti,ab,kw
24. oxazolam':ti,ab,kw
25. flutazolam':ti,ab,kw
26. etizolam':ti,ab,kw
27. mexazolam':ti,ab,kw
28. clotiazepam':ti,ab,kw
29. tofisopam':ti,ab,kw
30. 8 OR 9 OR 10 OR 11 OR 12 OR 13 OR 14 OR 15 OR 16 OR 17 OR 18 OR 19 OR 20 OR 21 OR 22 OR 23 OR 24 OR 25 OR 26 OR 27 OR 28
31. cognitive behavioral therapy'/exp
32. cognitive therapy'/exp
33. behavior therapy'/exp
34. behaviour therapy':ti,ab,kw
35. behavior':ti,ab,kw AND 'therapy':ti,ab,kw
36. behavior therapy':ti,ab,kw
37. cognitive':ti,ab,kw AND 'therapy':ti,ab,kw
38. cognitive therapy':ti,ab,kw
39. cognitive behaviour therapy':ti,ab,kw
40. cognitive':ti,ab,kw AND 'behavior':ti,ab,kw AND 'therapy':ti,ab,kw
41. cognitive behavior therapy':ti,ab,kw
42. 31 OR 32 OR 33 OR 34 OR 35 OR 36 OR 37 OR 38 OR 39 OR 40 OR 41
43. abstinen*':ti,ab,kw
44. abstain*':ti,ab,kw
45. cessat*':ti,ab,kw
46. detox*':ti,ab,kw
47. discontinu*':ti,ab,kw
48. reduce*':ti,ab,kw
49. reducing':ti,ab,kw
50. reduct*':ti,ab,kw
51. stop*':ti,ab,kw
52. taper*':ti,ab,kw
53. withdraw*':ti,ab,kw
54. substitut*':ti,ab,kw
55. 43 OR 44 OR 45 OR 46 OR 47 OR 48 OR 49 OR 50 OR 51 OR 52 OR 53 OR 54
56. 7 AND 30
57. 42 OR 55
58. 56 AND 57
59. crossover procedure':de OR 'double-blind procedure':de OR 'randomized controlled trial':de OR 'single-blind procedure':de OR random*:de,ab,ti OR factorial*:de,ab,ti OR crossover*:de,ab,ti OR ((cross NEXT/1 over*):de,ab,ti) OR placebo*:de,ab,ti OR ((doubl* NEAR/1 blind*):de,ab,ti) OR ((singl* NEAR/1 blind*):de,ab,ti) OR assign*:de,ab,ti OR allocat*:de,ab,ti OR volunteer*:de,ab,ti
60. 58 AND 59
61. 60 AND [embase]/lim NOT ([embase]/lim AND [medline]/lim)

**ClicalTrials.gov**

1. anxiety disorder [condition or disease]
2. cognitive behavioral therapy [condition or disease]
3. benzodiazepine [Other terms]
4. 1 AND 2 AND 3
