# Supplementary material for: Does cognitive behavioral therapy for anxiety disorders assist the discontinuation of benzodiazepines among patients with anxiety disorders? A systematic review and meta‐analysis
Source: Psychiatry Clin Neurosci. 2021 Feb 25;75(4):119–27. doi: 10.1111/pcn.13195 (PMC8048602; doi:10.1111/pcn.13195)
Supplement: Supplementary file 2 — Table S2. List of excluded articles. [file PCN-75-119-s001.docx]

**Supporting Information**

**Supporting Table S2. List of excluded articles**

| Study | Reason for exclusion |
| --- | --- |
| 1. A. Ataoglu 2000 (1) | Not appropriate w.r.t. patient sample (less than 80% taking benzodiazepines); Not appropriate w.r.t. intervention (CBT vs alprazolam) |
| 1. A. L. Granoff 2014 (2) | Not RCT (Letter to the Editor) |
| 1. A. Miretzky 1992 (3) | Full text not available (conference presentation); Not appropriate w.r.t. intervention (imipramine and CBT vs fluvoxamine and CBT) |
| 1. A. N. Niles 2014 (4) | Not appropriate w.r.t. patient sample (Not taking benzodiazepines or taking benzodiazepines for less than a month) |
| 1. A. Y. Takriti 1989 (5) | Not appropriate w.r.t. patient sample (less than 80% taking benzodiazepines); Not appropriate w.r.t. intervention (CBT vs alprazolam) |
| 1. B. Bandelow 2013 (6) | Not RCT (Review) |
| 1. B. L. Rollman 2018 (7) | Not appropriate w.r.t. patient sample (less than 80% taking benzodiazepines); Not appropriate w.r.t. diagnosis (not using operational diagnostic criteria) |
| 1. B. Milrod 2016 (8) | Not appropriate w.r.t. patient sample (less than 80% taking benzodiazepines) |
| 1. C. A. van Boeijen 2005 (9) | Not appropriate w.r.t. patient sample (Unknown percentage of benzodiazepine users); Not appropriate w.r.t. intervention (CBT vs manual vs guidelines) |
| 1. C. K. Gale 2013 (10) | Not RCT (Review) |
| 1. C. S. Gelernter 1991 (11) | Not appropriate w.r.t. patient sample (less than 80% taking benzodiazepines); Not appropriate w.r.t. intervention (group CBT treatment program with pharmacotherapy with alprazolam, phenelzine sulfate, or pill‐placebo plus instructions for self‐directed exposure to phobic stimuli) |
| 1. D. F. Klein 1993 (12) | Not RCT (Correspondence) |
| 1. E. de Beurs 1999 (13) | Not appropriate w.r.t. patient sample (less than 80% taking benzodiazepines); Not appropriate w.r.t. intervention ((i) fluvoxamine combined with exposure; (ii) placebo medication plus exposure; (iii) psychological panic management plus exposure; and (iv) exposure alone) |
| 1. E. E. Gorenstein 2005 (14) | Not appropriate w.r.t. patient sample (less than 80% taking benzodiazepines) |
| 1. E. Echeburua 2006 (15) | Not appropriate w.r.t. diagnosis (mixed anxiety depression) |
| 1. F. Akbarian 2015 (16) | Not appropriate w.r.t. patient sample (less than 80% taking benzodiazepines); Not appropriate w.r.t. diagnosis (PTSD) |
| 1. G. Crouch 1988 (17) | Not RCT; Not appropriate w.r.t. diagnosis (tranquilizer dependent patients) |
| 1. G. K. Brown 1997 (18) | Not appropriate w.r.t. patient sample (less than 80% taking benzodiazepines); Not appropriate w.r.t. intervention (focused cognitive therapy (FCT) vs standard cognitive therapy (SCT)) |
| 1. H. Cappell 1987 (19) | Not appropriate w.r.t. diagnosis (chronic user of benzodiazepine); Not appropriate w.r.t. intervention (behavioral therapy plus diazepam vs behavioral therapy plus placebo) |
| 1. H. Su 2007 (20) | Not appropriate w.r.t. diagnosis (PTSD) |
| 1. H. V. Curran 1994 (21) | Not appropriate w.r.t. patient sample (Unknown percentage of benzodiazepine users); Not appropriate w.r.t. intervention {(i) alprazolam and live exposure; (ii) alprazolam and relaxation; (iii) placebo and live exposure; and (iv) placebo and relaxation) |
| 1. J. Cottraux 2008 (22) | Not appropriate for patient sample (less than 80% taking benzodiazepines); Not appropriate for diagnosis (PTSD); Not appropriate for intervention (CBT or Rogerian supportive therapy) |
| 1. J. D. Roache 1999 (23) | No full text (monograph series of National Institute on Drug Abuse); Not appropriate w.r.t. diagnosis (Unknown based on operational diagnostic criteria) |
| 1. J. J. Arch 2007 (24) | Not appropriate w.r.t. patient sample (less than 80% taking benzodiazepines); Not appropriate w.r.t. intervention (group CBT) |
| 1. J. M. Newby 2014 (25) | Not appropriate w.r.t. patient sample (less than 80% taking benzodiazepines) |
| 1. J. Prasko 2004 (26) | Not appropriate w.r.t. patient sample (Unknown percentage of benzodiazepine users); Not appropriate w.r.t. intervention (CBT vs antidepressant) |
| 1. J. Prasko 2011 (27) | Not RCT; Not appropriate w.r.t. diagnosis (less than 80% anxiety disorder) |
| 1. J. S. Klosko 1988 (28) | Not appropriate w.r.t. patient sample (less than 80% taking benzodiazepines) |
| 1. J. S. Klosko 1990 (29) | Not appropriate w.r.t. patient sample (less than 80% taking benzodiazepines); |
| 1. J. S. Klosko 1994 (30) | Not appropriate w.r.t. patient sample (less than 80% taking benzodiazepines); |
| 1. J. V. Olthuis 2015 (31) | Not RCT (study protocol) |
| 1. J. Wardle 1994 (32) | Not appropriate w.r.t. patient sample (less than 80% taking benzodiazepines); Not appropriate w.r.t. intervention (exposure with diazepam vs exposure with placebo) |
| 1. K. Mugunthan 2011 (33) | Not RCT (systematic review) |
| 1. K. O'Connor 2008 (34) | Not appropriate w.r.t. intervention (group CBT) |
| 1. M. Başoğlu 1994 (35) | Not appropriate w.r.t. patient sample (less than 80% taking benzodiazepines) |
| 1. M. Başoğlu 1994 (36) | Not appropriate w.r.t. patient sample (less than 80% taking benzodiazepines) |
| 1. M. Başoğlu 1994 (37) | Not appropriate w.r.t. patient sample (less than 80% taking benzodiazepines) |
| 1. M. Başoğlu 1997 (38) | Not appropriate w.r.t. patient sample (less than 80% taking benzodiazepines) |
| 1. M. D. Auerbach 1997 | Full text not available (Conference presentation); Not appropriate w.r.t. intervention (CBT and alprazolam-XR vs CBT and placebo) |
| 1. M. E. Addis 2006 (39) | Not RCT (Observational study) |
| 1. M. Fux 1995 | Not appropriate w.r.t. patient sample (less than 80% taking benzodiazepines) |
| 1. M. G. Craske 1991 (40) | Not RCT (Mini Review) |
| 1. M. Ociskova 2016 (41) | Not RCT |
| 1. M. W. Otto 1993 (42) | Not appropriate w.r.t. intervention (taper and group CBT vs taper alone); Not appropriate w.r.t. diagnosis (not using operational diagnostic criteria) |
| 1. M. W. Otto 2005 (43) | Not RCT (Review) |
| 1. N. B. Schmidt 2005 (44) | Not appropriate w.r.t. patient sample (less than 80% taking benzodiazepines); Not appropriate w.r.t. intervention (group CBT) |
| 1. N. B. Schmidt 1997 (45) | Not appropriate w.r.t. patient sample (less than 80% taking benzodiazepines); Not appropriate w.r.t. intervention (group CBT) |
| 1. N. B. Schmidt 2002 (46) | Not appropriate w.r.t. patient sample (Unknown percentage of benzodiazepine users); Not appropriate w.r.t. intervention (group CBT) |
| 1. N. M. Bakhshani 2007 (47) | Not appropriate w.r.t. patient sample (including age < 18); Not appropriate w.r.t. patient sample (Unknown percentage of benzodiazepine users) |
| 1. N. M. Simon 2009 (48) | Not appropriate w.r.t. patient sample (less than 80% taking benzodiazepines) |
| 1. P. P. Roy-Byrne 2005 (49) | Not appropriate w.r.t. patient sample (Unknown percentage of benzodiazepine users) |
| 1. P. Tyrer 1993 (50) | Not appropriate w.r.t. patient sample (less than 80% taking benzodiazepines); Not appropriate w.r.t. diagnosis (including dysthymic disorder) |
| 1. P. Tyrer 1988 (51) | Not appropriate w.r.t. patient sample (less than 80% taking benzodiazepines); Not appropriate w.r.t. diagnosis (including dysthymic disorder) |
| 1. R. C. Durham 1999 (52) | Not RCT |
| 1. R. C. O. Voshaar 2001 (53) | Full text not available (Conference presentation); Not appropriate w.r.t. diagnosis (chronic benzodiazepine user) |
| 1. T. J. Bruce 1999 (54) | Not RCT |
| 1. T. J. Bruce 1995 (55) | Not RCT |
| 1. W. Katon 2006 (56) | Not appropriate w.r.t. patient sample (Unknown percentage of benzodiazepine users); Not appropriate w.r.t. intervention (combined CBT and pharmacotherapy vs treatment as usual) |
| 1. W. R. Lindsay 1987 (57) | Not appropriate w.r.t. patient sample (less than 80% taking benzodiazepines); Not appropriate w.r.t. diagnosis (not using operational diagnostic criteria) |
| 1. Y. H. Choi 2005 (58) | Not appropriate w.r.t. patient sample (less than 80% taking benzodiazepines): Not appropriate w.r.t. intervention (four sessions vs 12 sessions group experimental cognitive therapy) |
| 1. Y. W. Kim 2009 (59) | Not RCT |
| 1. Y. Zhang 2002 (60) | Not appropriate w.r.t. patient sample (less than 80% taking benzodiazepines) |

ACT= acceptance and commitment therapy, ART= applied relaxation training, CBT= cognitive behavioral therapy, PFPP= panic-focused psychodynamic psychotherapy, PTSD= post-traumatic stress disorder, RCT= randomized controlled trial, w.r.t.= with respect to

**References**

1. Ataoglu A, Ozkan M, Tutkun H, Maras A. Alprazolam and cognitive behavior therapy in treatment of panic disorder. Turkish Journal of Medical Sciences. 2000;30(2):167‐71.

2. Granoff AL. The Impact of Benzodiazepine Management in the Randomized, Double-Blind Evaluation ofd-Cycloserine or Alprazolam Combined With Virtual Reality Exposure Therapy. American Journal of Psychiatry. 2014;171(11):1222.

3. Miretzky A, Horn R, Koehler K, Moller HJ. Combination of alprazolam, antidepressive drugs and cognitive behavior therapy in the treatment of panic disorder. Clinical Neuropharmacology. 1992;15(1 Pt B):536.

4. Niles AN, Burklund LJ, Arch JJ, Lieberman MD, Saxbe D, Craske MG. Cognitive Mediators of Treatment for Social Anxiety Disorder: Comparing Acceptance and Commitment Therapy and Cognitive-Behavioral Therapy. Behavior Therapy. 2014;45(5):664-77.

5. Takriti AY, Zakaria Z. Panic disorder: alprazolam versus cognitive therapy. Arab Journal of Psychiatry. 1989;1(1):12‐21.

6. Bandelow B, Boerner RJ, Kasper S, Linden M, Wittchen H-U, Möller H-J. The Diagnosis and Treatment of Generalized Anxiety Disorder. Deutsches Aerzteblatt Online. 2013;110(17).

7. Rollman BL, Herbeck Belnap B, Abebe KZ, Spring MB, Rotondi AJ, Rothenberger SD, et al. Effectiveness of Online Collaborative Care for Treating Mood and Anxiety Disorders in Primary Care. JAMA Psychiatry. 2018;75(1):56-64.

8. Milrod B, Chambless DL, Gallop R, Busch FN, Schwalberg M, McCarthy KS, et al. Psychotherapies for panic disorder: a tale of two sites. Journal of Clinical Psychiatry. 2016;77(7):927-35.

9. van Boeijen CA, van Oppen P, van Balkom AJ, Visser S, Kempe PT, Blankenstein N, et al. Treatment of anxiety disorders in primary care practice: a randomised controlled trial. British Journal of General Practice. 2005;55(519):763-9.

10. Gale CK, Millichamp J. Generalized Anxiety Disorder. American Family Physician. 2013;87(2):122-4.

11. Gelernter CS, Uhde TW, Cimbolic P, Arnkoff DB, Vittone BJ, Tancer ME, et al. Cognitive-behavioral and pharmacological treatments of social phobia. A controlled study. Archives of General Psychiatry. 1991;48(10):938-45.

12. Klein DF. Panic disorder with agoraphobia. British Journal of Psychiatry. 1993;163(6):835-6.

13. Beurs E, Balkom AJLM, Dyck RV, Lange A. Long-term outcome of pharmacological and psychological treatment for panic disorder with agoraphobia: a 2-year naturalistic follow-up. Acta Psychiatrica Scandinavica. 1999;99(1):59-67.

14. Gorenstein EE, Kleber MS, Mohlman J, Dejesus M, Gorman JM, Papp LA. Cognitive-behavioral therapy for management of anxiety and medication taper in older adults. American Journal of Geriatric Psychiatry. 2005;13(10):901-9.

15. Echeburua E, Salaberria K, De Corral P, Cenea R, Berasategui T. Treatment of mixed anxiety-depression disorder: long-term outcome. Behavioural and Cognitive Psychotherapy. 2006;34(1):95‐101.

16. Akbarian F, Bajoghli H, Haghighi M, Kalak N, Holsboer-Trachsler E, Brand S. The effectiveness of cognitive behavioral therapy with respect to psychological symptoms and recovering autobiographical memory in patients suffering from post-traumatic stress disorder. Neuropsychiatric Disease and Treatment. 2015;11:395-404.

17. Crouch G, Robson M, Hallstrom C. Benzodiazepine dependent patients and their psychological treatment. Progress in Neuro-psychopharmacology and Biological Psychiatry. 1988;12(4):503-10.

18. Brown GK, Beck AT, Newman CF, Beck JS, Tran GQ. A Comparison of Focused and Standard Cognitive Therapy for Panic Disorder. Journal of Anxiety Disorders. 1997;11(3):329-45.

19. Cappell H, Busto U, Kay G, Naranjo C, Sellers E, Sanchez-Craig M. Drug deprivation and reinforcement by diazepam in a dependent population. Psychopharmacology. 1987;91(2):154-60.

20. Su H, Wang JT, Lou ZS, Lu HT. Cognitive-exposure therapy for post-traumatic stress disorder. Journal of Clinical Rehabilitative Tissue Engineering Research. 2007;11(39):7783‐6.

21. Curran HV, Bond A, O'Sullivan G, Bruce M, Marks I, Lelliot P, et al. Memory functions, alprazolam and exposure therapy: a controlled longitudinal study of agoraphobia with panic disorder. Psychological Medicine. 1994;24(4):969-76.

22. Cottraux J, Note I, Yao SN, de Mey-Guillard C, Bonasse F, Djamoussian D, et al. Randomized controlled comparison of cognitive behavior therapy with Rogerian supportive therapy in chronic post-traumatic stress disorder: a 2-year follow-up. Psychotherapy and Psychosomatics. 2008;77(2):101-10.

23. Roache JD, Oswald LM, Stanley MA, Creson DR, Shah NN. Effects of cognitive behavior therapy for anxiety on alprazolam self-medical behavior. NIDA research monograph. 1999:306.

24. Arch JJ, Craske MG. Implications of naturalistic use of pharmacotherapy in CBT treatment for panic disorder. Behaviour Research and Therapy. 2007;45(7):1435-47.

25. Newby JM, Williams AD, Andrews G. Reductions in negative repetitive thinking and metacognitive beliefs during transdiagnostic internet cognitive behavioural therapy (iCBT) for mixed anxiety and depression. Behaviour Research and Therapy. 2014;59:52-60.

26. Prasko J, Horacek J, Zalesky R, Kopecek M, Novak T, Paskova B, et al. The change of regional brain metabolism (18FDG PET) in panic disorder during the treatment with cognitive behavioral therapy or antidepressants. Neuro Endocrinology Letters. 2004;25(5):340-8.

27. Prasko J, Latalova K, Diveky T, Grambal A, Kamaradova D, Velartova H, et al. Panic disorder, autonomic nervous system and dissociation - changes during therapy. Neuro Endocrinology Letters. 2011;32(5):641-51.

28. Klosko JS. Comparison of alprazolam and cognitive-behavior therapy in treatment of panic disorder. Dissertation Abstracts International. 1988;49(5‐b):1945.

29. Klosko JS, Barlow DH, Tassinari R, Cerny JA. A comparison of alprazolam and behavior therapy in treatment of panic disorder. Journal of Consulting and Clinical Psychology. 1990;58(1):77-84.

30. Klosko JS, Barlow DH, Tassinari R, Cerny JA. A comparison of alprazolam and behavior therapy in treatment of panic disorder. Journal of Psychotherapy Practice and Research. 1994;3(2):163-79.

31. Olthuis JV, Watt MC, Mackinnon SP, Stewart SH. Telephone-delivered cognitive behavioral therapy for high anxiety sensitivity: a randomized controlled trial. Journal of Consulting and Clinical Psychology. 2015;82(6):1005‐22.

32. Wardle J, Hayward P, Higgitt A, Stabl M, Blizard R, Gray J. Effects of concurrent diazepam treatment on the outcome of exposure therapy in agoraphobia. Behaviour Research and Therapy. 1994;32(2):203‐15.

33. Mugunthan K, McGuire T, Glasziou P. Minimal interventions to decrease long-term use of benzodiazepines in primary care: a systematic review and meta-analysis. British Journal of General Practice. 2011;61(590):e573-8.

34. O'Connor K, Marchand A, Brousseau L, Aardema F, Mainguy N, Landry P, et al. Cognitive–behavioural, pharmacological and psychosocial predictors of outcome during tapered discontinuation of benzodiazepine. Clinical Psychology & Psychotherapy. 2008;15(1):1-14.

35. Basoglu M, Marks IM, Kilic C, Brewin CR, Swinson RP. Alprazolam and exposure for panic disorder with agoraphobia. Attribution of improvement to medication predicts subsequent relapse. British Journal of Psychiatry. 1994;164(5):652-9.

36. Basoglu M, Marks IM, Kilic C, Swinson RP, Noshirvani H, Kuch K, et al. Relationship of panic, anticipatory anxiety, agoraphobia and global improvement in panic disorder with agoraphobia treated with alprazolam and exposure. British Journal of Psychiatry. 1994;164(5):647-52.

37. Basoglu M, Marks IM, Swinson RP, Noshirvani H, O'Sullivan G, Kuch K. Pre-treatment predictors of treatment outcome in panic disorder and agoraphobia treated with alprazolam and exposure. Journal of Affective Disorders. 1994;30(2):123-32.

38. Basoglu M, Marks I, Livanou M, Swinson R. Double-blindness procedures, rater blindness, and ratings of outcome. Observations from a controlled trial. Archives of General Psychiatry. 1997;54(8):744-8.

39. Addis ME, Hatgis C, Cardemil E, Jacob K, Krasnow AD, Mansfield A. Effectiveness of cognitive-behavioral treatment for panic disorder versus treatment as usual in a managed care setting: 2-Year follow-up. Journal of Consulting and Clinical Psychology. 2006;74(2):377‐85.

40. Craske MG. Models and treatment of panic: behavioural therapy of panic. Journal of Cognitive Psychotherapy: An International Quarterly. 1991;5(3):199‐214.

41. Prasko J, Ociskova M, Latalova K, Kamaradova D, Grambal A. Psychological factors and treatment effectiveness in resistant anxiety disorders in highly comorbid inpatients. Neuropsychiatric Disease and Treatment. 2016;12:1539-51.

42. Otto MW, Pollack MH, Sachs GS, Reiter SR, Meltzer-Brody S, Rosenbaum JF. Discontinuation of benzodiazepine treatment: efficacy of cognitive-behavioral therapy for patients with panic disorder. American Journal of Psychiatry. 1993;150(10):1485‐90.

43. Otto MW, Bruce SE, Deckersbach T. Benzodiazepine use, cognitive impairment, and cognitive-behavioral therapy for anxiety disorders: issues in the treatment of a patient in need. Journal of Clinical Psychiatry. 2005;66 Suppl 2:34-8.

44. Schmidt NB, Smith JD. Do medications matter in the context of cognitive behavior therapy for panic disorder? Journal of Cognitive Psychotherapy. 2005;19(4):347-54.

45. Schmidt NB, Trakowski JH, Staab JP. Extinction of panicogenic effects of a 35% CO2 challenge in patients with panic disorder. Journal of Abnormal Psychology. 1997;106(4):630‐8.

46. Schmidt NB, Wollaway-Bickel K, Trakowski JH, Santiago HT, Vasey M. Antidepressant discontinuation in the context of cognitive behavioral treatment for panic disorder. Behaviour Research and Therapy. 2002;40(1):67-73.

47. Bakhshani NM, Lashkaripour K, Sadjadi SA. Effectiveness of short term cognitive behavior therapy in patients with generalized anxiety disorder. Journal of Medical Sciences (Taipei, Taiwan). 2007;7(7):1076‐81.

48. Simon NM, Otto MW, Worthington JJ, Hoge EA, Thompson EH, Lebeau RT, et al. Next-step strategies for panic disorder refractory to initial pharmacotherapy: a 3-phase randomized clinical trial. Journal of Clinical Psychiatry. 2009;70(11):1563‐70.

49. Roy-Byrne PP, Craske MG, Stein MB, Sullivan G, Bystritsky A, Katon W, et al. A Randomized Effectiveness Trial of Cognitive-Behavioral Therapy and Medication for Primary Care Panic Disorder. Archives of General Psychiatry. 2005;62(3):290-8.

50. Tyrer P, Seivewright N, Ferguson B, Murphy S, Johnson AL. The Nottingham study of neurotic disorder. Effect of personality status on response to drug treatment, cognitive therapy and self-help over two years. British Journal of Psychiatry. 1993;162(2):219‐26.

51. Tyrer P, Seivewright N, Murphy S, Ferguson B, Kingdon D, Barczak P, et al. The Nottingham study of neurotic disorder: comparison of drug and psychological treatments. Lancet (London, England). 1988;2(8605):235‐40.

52. Durham RC, Fisher PL, Treliving LR, Hau CM, Richard K, Stewart JB. One year follow-up of cognitive therapy, analytic psychotherapy and anxiety management training for generalized anxiety disorder: symptom change, medication usage and attitudes to treatment. Behavioural and Cognitive Psychotherapy. 1999;27(1):19‐35.

53. Voshaar RCO, Gorgels WDJ, Mol AJ, Balkom A, Breteler RM, Zitman FG. Benzoredux study: a two-phase approach to reduce chronic benzodiazepine use. 2001 Annual Meeting of the American Psychiatric Association; 2001 May 5-10; New Orleans; LA, USA. 2001.

54. Bruce TJ, Spiegel DA, Hegel MT. Cognitive-behavioral therapy helps prevent relapse and recurrence of panic disorder following alprazolam discontinuation: a long-term follow-up of the Peoria and Dartmouth studies. Journal of Consulting and Clinical Psychology. 1999;67(1):151‐6.

55. Bruce TJ, Spiegel DA, Gregg SF, Nuzzarello A. Predictors of alprazolam discontinuation with and without cognitive behavior therapy in panic disorder. American Journal of Psychiatry. 1995;152(8):1156‐60.

56. Katon W, Russo J, Sherbourne C, B. Stein M, Craske M, Fan M-Y, et al. Incremental cost-effectiveness of a collaborative care intervention for panic disorder. Psychological Medicine. 2006;36(3):353-63.

57. Lindsay WR, Gamsu CV, McLaughlin E, Hood EM, Espie CA. A controlled trial of treatments for generalized anxiety. British Journal of Clinical Psychology. 1987;26 ( Pt 1):3‐15.

58. Choi Y-H, Vincelli F, Riva G, Wiederhold BK, Lee J-H, Park K-H. Effects of Group Experiential Cognitive Therapy for the Treatment of Panic Disorder with Agoraphobia. CyberPsychology & Behavior. 2005;8(4):387-93.

59. Kim YW, Lee S-H, Choi TK, Suh SY, Kim B, Kim CM, et al. Effectiveness of mindfulness-based cognitive therapy as an adjuvant to pharmacotherapy in patients with panic disorder or generalized anxiety disorder. Depression and Anxiety. 2009;26(7):601-6.

60. Zhang Y, Young D, Lee S, Zhang H, Xiao Z, Hao W, et al. Chinese Taoist Cognitive Psychotherapy in the Treatment of Generalized Anxiety Disorder in Contemporary China. 2002;39(1):115-29.
